# Screening of Generalized Anxiety Disorder in Patients with Epilepsy: Using a Valid and Reliable Indonesian Version of Generalized Anxiety Disorder-7 (GAD-7)

**DOI:** 10.1155/2019/5902610

**Published:** 2019-06-02

**Authors:** Astri Budikayanti, Andira Larasari, Khamelia Malik, Zakiah Syeban, Luh Ari Indrawati, Fitri Octaviana

**Affiliations:** ^1^Department of Neurology, Faculty of Medicine, Universitas Indonesia/Cipto Mangunkusumo General Hospital, Jakarta, Indonesia; ^2^Department of Psychiatry, Faculty of Medicine, Universitas Indonesia/Cipto Mangunkusumo General Hospital, Jakarta, Indonesia

## Abstract

**Introduction:**

Generalized anxiety disorder (GAD) is one of the most common types of anxiety disorder in epilepsy population, comprising 21.9%, that would further impair patients' quality of life. Generalized Anxiety Disorder-7 (GAD-7) is the only screening tool for GAD that has been validated in patients with epilepsy (PWE). It is a self-reporting instrument that can be completed in less than three minutes; hence, its usage is appropriate in primary healthcare and neurology outpatient clinic. This study aimed to obtain a valid and reliable Indonesian version of GAD-7, assess its accuracy, and finally evaluate the prevalence of GAD in Indonesian PWE along with its contributing factors.

**Methods:**

A cross-sectional study was conducted in Cipto Mangunkusumo General Hospital, Jakarta. The GAD-7 was translated and adapted using World Health Organization (WHO) steps. Validity, reliability, test-retest reliability, and diagnostic accuracy were evaluated. Then, epilepsy outpatients were screened for GAD using the Indonesian version of GAD-7.

**Results:**

Internal validity and reliability for Indonesian version of GAD-7 were satisfactory with validity coefficient of 0.648 to 0.800 (p<0.01) and Cronbach's alpha value of 0.867. The best cutoff value to detect GAD in Indonesian PWE was >6 with the sensitivity, specificity, negative predictive value, and positive predictive value of 100%, 84.4%, 100%, and 55.8%, respectively. ROC analysis showed the area under the curve of 0.98 (95% CI: 0.96–0.99). The total subjects screened with the validated Indonesian version of GAD-7 were 146, and 49% were screened as having GAD. Sociodemographic and clinical characteristics had no statistically significant association with the presence of GAD.

**Conclusion:**

The Indonesian version of GAD-7 was a valuable screening tool to detect GAD in PWE. GAD was screened in a quite high proportion of PWE. Sociodemographic and clinical characteristics were not proven to play role in its development.

## 1. Introduction

Patients with epilepsy (PWE) are more susceptible to psychiatric disorders compared to general population [1]. Aside from the psychological aspects as a consequence of this disorder, neurobiological causes including neurotransmitter imbalance and anatomical abnormalities, along with the use of antiepileptic drugs (AEDs), may also contribute to the high incidence of psychiatric disorder in PWE. Limbic system circuitry disturbance in temporal lobe epilepsy also plays a role in psychiatric disorders, especially anxiety and depression [2, 3]. Anxiety disorder is one of the most common psychiatric problems in PWE (10-25%), subsequent to depression (24-74%) [4, 5]. The comorbidity of anxiety disorder and depression occur in a significant number of PWE. Seventy-three percent of PWE with depression also fulfills the criteria for anxiety disorder [6]. The incidence of anxiety disorder alone from a multicenter study in United States was reported as high as 30.4% in adult chronic epilepsy.

Moreover, the prevalence of anxiety disorder in PWE is twofold higher than the general population, especially for uncontrolled epilepsy, and is identified as a risk factor for drug-refractory epilepsy [7–9]. It is also associated with stigmatization, suicidal ideation, and impaired patients' quality of life.

Anxiety disorder is a group of mental disorders characterized by excessive feelings of anxiety and fear that continually manifest, accompanied by autonomic symptoms of palpitation, over perspiration, as well as uncomfortable feeling in the stomach or chest [10–12]. Generalized anxiety disorder (GAD) refers to the presence of persistent anxiety, unlimited to certain environmental conditions, and will last for at least 6 months. The patients will experience fear even in common problems at work, health, financial issues, or family. They usually worry about the occurrences of epileptic seizure, severity of their illness, and the emerging of complications. This condition coexists with somatic symptoms like exhaustion, insomnia, and difficulty in concentrating [10, 13].

Early detection of anxiety disorder, especially GAD, becomes crucial to maintain the psychosocial function in PWE. However, the strategy of using structured interview is challenging due to its long duration, high cost, and the need of psychiatrist or other trained medical personnel to administer the test. A sensitive, easy, and practical screening method that can be applied in outpatient clinic is highly needed, but to date there is no instrument to be used specifically in Indonesian PWE [8, 10, 14].

Generalized Anxiety Disorder-7 (GAD-7) is the only screening instrument to detect GAD that has been validated in PWE with good internal consistency [15]. Compared to other screening instruments such as Mini International Neuropsychiatric Interview-International Classification of Diseases 10 (MINI ICD-10), GAD-7 is considered to be easier. It is a self-reporting instrument and consists of short questions that can be completed in less than 3 minutes. Therefore, the presence of psychiatrists or other trained medical personnel in the screening process is no longer mandatory [14, 16, 17].

GAD-7 questionnaire is recommended for PWE since it has no question regarding somatic symptoms that could be misinterpreted as the adverse effects of AED, cognitive impairments, or neurological disorders due to epilepsy [18]. This study aimed to obtain a valid and reliable Indonesian version of GAD-7 as a screening instrument to detect GAD in PWE.

## 2. Methods

This study was conducted in epilepsy outpatient clinic, Cipto Mangunkusumo General Hospital, in August 2015. Ethical clearance was granted by the ethical committee of Faculty of Medicine, Universitas Indonesia. The GAD-7 questionnaire can be downloaded from www.phqscreeners.com without needing any permission to be copied, translated, and distributed [19]. Nevertheless, the study team decided to inform Dr. Robert L. Spitzer and Dr. Kurt Kroenke as the GAD-7 developers regarding the research plan.

### 2.1. Translation and Validation Phase

Translation and adaptation of GAD-7 into Bahasa Indonesia was performed using the cultural adaptation steps by WHO [20]. The content validity of the questionnaire was then evaluated by the relevance of each question with the GAD diagnostic criteria by five neurologists and five psychiatrists. Each person qualitatively scored the questions according to Likert scale of 1 (irrelevant); 2 (relevant); and 3 (very relevant). The validity coefficient was calculated using Martuzua method [21].

Internal validity and reliability test were performed in 30 subjects who were consecutively collected, to maximize the accuracy. Subjects who fulfilled the inclusion criteria of age >18 years old, being diagnosed with epilepsy for at least 6 months, being capable to communicate, read, and write in Bahasa Indonesia as well as to answer independently, being at stable dose of AED between the first and second visit, and agreeing to participate were recruited to the study. The exclusion criteria were having hearing problem, severe visual impairment, mental retardation, history of neurological, psychiatric, or severe medical problems, and history of alcohol use or drug abuse. The diagnosis of mental retardation was assessed with a thorough neurobehavior examination. Self-reported GAD-7 assessments and retests were done between 1 and 2 weeks later.

Internal validity was analyzed using Spearman correlation coefficient to find out the correlation between each question and the total score of the questionnaire. If the coefficient approached the value of 1, a stronger correlation was assumed, with the classification as follows: (1) 0.0 - <0.02 suggests a very weak correlation, (2) 0,2 - <0,4 suggests weak correlation, (3) 0,4 - <0,6 indicates moderate correlation, (4) 0,6 - <0,8 depicts strong correlation, and (5) 0,8 – 1,0 depicts a very strong correlation.

In this study, we performed both internal and external reliability test. Internal reliability test was performed using* Cronbach's alpha *statistical analysis. A good reliability could be depicted from* Cronbach's alpha* value of more than 0.7. Furthermore, for the external reliability analysis, we compared the correlation coefficients and* Cronbach's alpha* value was obtained from the first and retest examination.

### 2.2. Prevalence and Diagnostic Study

For the diagnostic accuracy assessment of the questionnaire, we collected 146 subjects consecutively with the same inclusion and exclusion criteria as mentioned above. Subjects that were eligible to participate and had signed a written informed consent were assessed using MINI ICD-10 as definitive tools to diagnose the presence of GAD and GAD-7 questionnaire as the new screening tools. Descriptive data were then presented in percentage, median, or mean as appropiate. A diagnostic test using 2x2 table with comparison to MINI ICD-10 as the gold standard was performed to calculate the sensitivity, specificity, negative predictive value (NPV), and positive predictive value (PPV).

A thorough history taking and physical examination were performed whereas the other data were collected from medical records, including electroencephalography (EEG) result, radiologic finding through magnetic resonance imaging (MRI) or computerized tomography (CT) scan data, and the current type of AED administered. Statistical analysis was performed using the SPSS 20.0. The data was subsequently arranged in a frequency distribution table or cross table in accordance with the study purpose. The *p* values that were less than 0.05 were considered statistically significant. Statistical analysis was performed using chi-square test, Fisher's exact test, and Kolmogorov-Smirnov test as an alternative to assess the association between sociodemographic and clinical characteristics with the occurrence of GAD.

### 2.3. Compliance with Ethics Guideline

Subjects in this study were recruited after gaining approval from the ethics committee and written informed consents for the study were obtained. The ethical approval was granted from Ethical Committee, Faculty of Medicine, Universitas Indonesia.

## 3. Results

### 3.1. Translation and Cultural Adaptation

The GAD-7 questionnaire was translated and culturally adapted according to the WHO steps. Modifications were made, including the additional time frame of “2 weeks” for each question and changing “half of the days” into “half of the 2 weeks.” Other minor linguistic adjustments were made along the adaptation process.

### 3.2. Validity and Reliability Analysis

The internal validation and reliability test were completed in 30 subjects, resulting in demographic features shown in Table 1. The content validity was assessed to see the relevance of each question by 10 experts that consisted of 5 consultants from the psychiatry department and 5 consultants from the epilepsy division of neurology department. Using the Martuzua method, the validity coefficient obtained was 0.847.

Internal validity test was analyzed using Spearman correlation showed that coefficients for the first examination fell between 0.648 and 0.800 and for the retest examination was between 0.680 and 0.832 (p value <0.01), indicating strong correlation for both test and retest. Regarding the internal reliability test, Cronbach's alpha values for the first and retest examination were 0.867 and 0.866, respectively. The reliability test for each “if an item was deleted” showed lower Cronbach's alpha values, albeit still above 0.700. Similar results were also observed in the retest examination.

Test-retest reliability comparing correlation coefficient and Cronbach's alpha value between the first and retest examination was 0.5%-10% for the correlation coefficient and 0.1% for Cronbach's alpha value.

### 3.3. Diagnostic Study

The valid and reliable Indonesian version of GAD-7 questionnaires was completed by 146 subjects with the median age of 36 ranging from 18 to 77. The majority of the subjects were male (54.1%), high school graduate (74.7%), unemployed (57.5%), and unmarried (52.1%) (Table 2).

Table 3 shows onset of epilepsy that were widely ranged from 1 year old up to 74 years old, also the duration of epilepsy from 6 months to 55 years. Seven of the 146 subjects had neither EEG nor radiologic data. Almost all subjects had focal onset seizure (98%); 69.1% originated from temporal lobe, and no lateralization was predominant. The most common etiology of epilepsy was structural with 54.8%; abnormality of hippocampal was not common. In terms of AED, most subjects (65.1%) were administered one type of AED with carbamazepine (31.5%) being the most common, followed by phenytoin (29.5%), valproic acid (28.8%), clobazam (21.2%), and phenobarbital (21.2%).

The sensitivity and specificity analysis resulted in >6 as the best cutoff value to detect GAD in PWE which yielded in highest sensitivity and specificity (Table 4). A diagnostic study was done to compare the result from GAD-7 and MINI ICD-10. From 2x2 table analysis, the sensitivity obtained was 100% with 84.4% specificity (Table 5). The negative predictive value was 100% and positive predictive value was 55.8%. The AUC value obtained was 0.98 (95%CI 0.96-0.99), indicating a very good prediction value of the GAD-7 questionnaire.

### 3.4. GAD Prevalence and Influencing Factors

Indonesian PWE that were screened to have concomitant GAD using Indonesian version of GAD-7 were 29%. Meanwhile, based on MINI-ICD 10 there were only 16.4% subjects who were diagnosed with GAD. The median score of GAD-7 in subjects with GAD was significantly higher than those with no GAD (p: 0.000) with 12(7-19) and 3(0-11), respectively (Table 6). There were no statistical differences between male and female predominance, educational level, and marital status in the occurrence of GAD. In subjects who were unemployed, GAD was more frequently found, even though it was not significant statistically.

In this study, there were only three subjects with generalized onset seizure and genetic etiology. Two out of three subjects were diagnosed with GAD. Meanwhile, in focal onset seizure, the proportion was only less than one-third subjects. There was no statistically significant association across all clinical characteristics with the presence of GAD. Nevertheless, in epilepsy with structural etiology, more subjects with other structural abnormality than hippocampal sclerosis/atrophy actually experienced GAD (25.6% vs 10.8%).

## 4. Discussion

A translation and cultural adaptation of the original GAD-7 version into Bahasa Indonesia are required when the measurement tools are to be used in the setting of an area with different cultural background. This process aims to pertain the validity of the instrument across different cultures [22, 23]. The original version of GAD-7 in English has been proven valid both in content and in structure, and it has been translated into several languages and cultures in Spain, Turkey, Malaysia, Portuguese, and Korea [15, 16, 24–28]. Most of the adapted version of GAD-7 was performed in general population, whereas those performed in PWE had only been reported in Korea and currently in Indonesia [15].

The content validity was evaluated by both psychiatrists and neurologists. The score obtained was 0.847, very close to 1 which indicated a good validity of GAD-7 in assessing the symptoms of GAD. On internal validity test, significant correlation was obtained from all questions (p<0.01), with correlation coefficient falling between 0.648 and 0.800, depicting a strong to very strong correlation. This result was comparable to a Korean study by Seo et al. (2014) whose observed correlation coefficients were 0.731 and 0.833 [15].

Internal reliability and consistency test resulted in Cronbach's alpha value of 0.876, which meant that the whole questionnaire was considered reliable. Reliability test for each “if an item was deleted” was evaluated and Cronbach's alpha values were lower, yet still above 0.700. Hence, we concluded that each question contributed to total Cronbach's alpha value. Similar results were also seen in the retest examination.

A high Cronbach's alpha value was also observed in Korean study with the value of 0.924 [15]. Moreover, a study in USA of the original version of GAD-7 also yielded a good value of 0.920 [16]. In Malaysia, a study performed in female general population gave Cronbach's alpha value of 0.740 [27]. Meanwhile, a study in Spain, Portugal, and Turkey, all in PWE, showed Cronbach's alpha value of 0.936, 0.880, and 0.852, respectively [24–26].

All reliability tests on GAD-7 from different countries and cultures showed good and very good results. Variability of Cronbach's alpha value resulted from variation in culture and subjects' characteristics. Multicenter studies conducted in Korea, USA, and Spain report higher Cronbach's alpha values due to heterogeneous samples [15, 16, 24]. A study in Malaysia had the smallest value since the subjects were homogenous, all female from one primary care clinic [27].

Test-retest reliability was assessed by comparing correlation coefficient and Cronbach's alpha value of each question from the first test and the retest. The difference in correlation coefficient was 0.5%-10%, while Cronbach's alpha value differed by only 0.1%. These small differences showed that GAD-7 was considered stable to be used in different time.

A study conducted in USA which included 591 subjects in general population had internal correlation coefficient of 0.830 which showed a high stability of GAD-7 [16]. Another study in Turkey, Portugal, and Spain resulted in the comparison between the first and retest examination of no significant difference [24–26].

Using GAD-7 questionnaire, 29% subjects were screened as having GAD, compared to only 16.4% subjects diagnosed using MINI ICD-10. Sensitivity and specificity test (with >6 as the cutoff) were 100% and 84.4%, consecutively. This result showed that 100% subjects with GAD were detected using the questionnaire and 84.4% patients without GAD were correctly excluded. Positive predictive value of 55.8% showed that only 55.8% of subjects detected as having GAD from the GAD-7 questionnaire were really having GAD by gold standard measurement; this depicted GAD-7 as having big false positive value. On the other hand, the negative predictive value of GAD-7 was 100%, depicting 0% false negative. The GAD-7 questionnaire was proposed as a screening tool, so it is important to have a high sensitivity value. Moreover, a significant difference in GAD-7 median score between GAD and non-GAD group (12 vs 3) supported the use of GAD-7 as a screening tool for GAD in PWE.

The sensitivity and specificity of Indonesian version of GAD-7 were comparable to a similar study by Seo et al. conducted in Korea, with the sensitivity and specificity of 92.2% and 89.1%, respectively, who also used >6 as the cutoff value [15]. This cutoff value was lower than those in general population. From study by Kroenke et al., the best cutoff value in detecting GAD in general population was 10 [17]. Plummer et al. in a systematic review and meta-analysis stated that a single cutoff value was not recommended; cutoff values ranging 7-10 fairly estimate the test accuracy. Thus clinicians may have different preferences [28]. Other studies to assess the diagnosis accuracy in PWE were performed by Micoulaud-Franchi in France and Tong in China with the best cutoff values obtained being >7 and >6, respectively. These variations were postulated to be based on the cultural differences in each country [29, 30].

The prevalence of GAD in this study was 29%. This number was higher than those in other countries, such as Canada with 22.8% PWE suffering from any anxiety disorder and 21% American PWE with GAD [1]. In several British studies, the prevalence of GAD also varied between 10 and 25% [31, 32]. In France, Micoulaud-Franchi et al. also found a high prevalence of GAD (33.8%), while Spain demonstrated lower number (6-8%) [24, 29]. In Asian countries such as Korea, the frequency of anxiety disorder in PWE was 21% using MINI-Plus 5.0.0 [15]. In Chinese population, GAD was diagnosed in 23.5% [30]. This wide variation across different countries, and even in the same country, may be caused by different tools in assessing GAD.

While no significant association was found between any clinical characteristics and the presence of GAD in PWE, the high prevalence of GAD in this study was suggested to be caused by the higher frequency of temporal lobe epilepsy (TLE) patients. This high frequency of TLE was also reported by Micoulaud-Franchi et al., which together with the high number of female in the study population contributed to the higher prevalence of GAD [29].

Of 146 subjects who participated in this study, most were unmarried and unemployed, with 52.1% and 57.5%, respectively, despite the subjects' mean age of 45.7 (17.4) which depicted a productive age. This high number of unmarried cases and being unemployed in PWE might be subjected to the bad stigma in society. In a study by Khairani A et al. in Indonesia, the general population consisting of family of PWE and lay people still had some misunderstanding regarding epilepsy and around 60% were reluctant to be married with PWE. Regarding employment, as many as 44% respondents objected to employ PWE [33]. A high number of unmarried statuses of PWE were also reported by several studies from different countries such as in Iran with only 27.3% and Brazil [34, 35]. Also, the high rate of employment was also seen in recent study by Lopes De Souza et al. in 2018 [36]. Even though high educational level was known to be protective against any form of anxiety disorder and depression in general population [37] and most subjects in our study finished high school, there was no significant difference in the prevalence of GAD across different educational levels. We also found that subjects who were unemployed had higher proportion of GAD compared to those who were employed (21.4% vs 9.7%) as can be seen in Table 1; in this study more than 50% of subjects were unemployed. This fact might contribute to the higher prevalence of GAD screened. Nevertheless, the association of employment and occurrence of GAD was not statistically significant as also shown by Bjelland I et al. study in general population and Seo et al. study in PWE [15, 37].

No significant association between any clinical characteristics and the presence of GAD in PWE was also found in Korean study by Seo et al. in 2014 and French study by Micoulaud-Franchi et al. in 2016 [15, 29]. A different result was found in a Chinese cohort by Tong et al. in which focal seizure with impaired awareness type, etiology, higher seizure frequency, and AED polytherapy significantly associated with the presence of GAD [35]. Furthermore, with respect to the etiology of epilepsy, Tong et al. reported that genetic etiology had the lowest frequency of GAD, while structural and unknown etiology were more frequent. In accordance to our study, structural etiology was the most frequent etiology. Other structural abnormalities were slightly higher than hippocampal sclerosis/atrophy, although the exact causes were not recorded. The presence of other comorbidities may contribute to a slightly higher frequency of GAD in other structural etiology groups.

The type of AED used by patients did not seem to correlate with the presence of GAD in this study. AED has long been known to have diverse psychotropic effects both negative and positive. In a report by Nadkarni et al. in 2005 it was discussed that AED such as levetiracetam was known to cause anxiety as its adverse effect. On the other hand, barbiturates and gabapentin were known to treat various forms of anxiety disorder [38]. In another report by Chen et al. in 2017, levetiracetam, lamotrigine, and tiagabine were known to cause higher rate of anxiety adverse effects compared to other AED [39]. Moreover, in a previously conducted study in our institution on the adverse effect of AED, it was shown that several adverse effects related to anxiety disorder were screened using LAEP questionnaire such as unsteadiness, restlessness, nervousness, and disturbed sleep. This could be associated with the most widely used AED in the study, which were carbamazepine and levetiracetam [40]. The lack of association of AED and the presence of GAD in this study need to be explored further in the future considering the duration of AED used and its dosage.

In this study, the association between sociodemographic and clinical characteristics with the presence of GAD was analyzed using the validated Indonesian version of GAD-7 questionnaire, to see whether certain characteristics might contribute. This approach was different from other previous studies which used the diagnosis made by MINI-ICD. We considered this as a limitation in our study. A different result might be obtained otherwise.

In summary, Indonesian version of GAD-7 questionnaire was considered to be an effective screening tool in detecting GAD in PWE since it is easy and self-reporting. There was no clinical characteristics found to be significant in increasing the risk of having GAD in PWE.

## Figures and Tables

**Table 1:** Subjects' demographic features for validation study (n=30).

Variable	n (%)/ mean ±SD
Gender	
Male	12 (40)
Female	18 (60)
Age Groups	
< 50 years old	16 (53,.3)
≥ 50 years old	14 (46.7)
Mean +/- SD	45.7 ± 17.40
Education	
Min. High School	15 (50)
Not finished High School	15 (50)

**Table 2:** Subjects' demographic characteristics (n=146).

Demographic Characteristics	n (%) / Median (min-max)
Age (years)	36 (18-77)
Gender	
Male	79 (54.1)
Female	67 (45.9)
Education	
Finished High School	109 (74.7)
Not Finished High School	37 (25.3)
Employment	
Employed	62 (42.5)
Unemployed	84 (57.5)
Marital Status	
Married	70 (47.9)
Unmarried	76 (52.1)

**Table 3:** Subjects' medical characteristics (n=146).

Medical Characteristics	n (%) / Median (min-max)
Onset of Epilepsy (year)	17.5 (1-74)
Duration of Epilepsy (year)	11 (0.5-55)
Seizure Frequency (times)	1 (0-52)
Seizure Type	
Focal Onset	143 (97.9)
Generalized Onset	3 (2.1)
Epileptic focality from EEG	
Right	61 (41.8)
Left	60 (41.1)
Focal Bilateral	15 (10.3)
General	3 (2.1)
No data	7 (4.8)
Epilepsy Syndrome (n=139)	
Temporal	96 (65.8)
Extratemporal	40 (27.4)
Generalized	3 (2.1)
No data	7 (4.8)
AED Administered	
1 AED	95 (65.1)
2 AED	40 (27.4)
≥ 3 AED	11 (7.5)
Etiology of Epilepsy	
Genetic	3 (2.1)
Unknown Etiology	63 (43.2)
Structural	80 (54.7)
(i) Hippocampal atrophy/sclerosis	37 (46.3)
(ii) Other Structural Abnormalities	43 (53.7)
Type of AED	
Valproate	42 (28.8%)
Carbamazepine	46 (31.5%)
Levetiracetam	10 (6.8%)
Phenytoin	43 (29.5%)
Clobazam	31 (21.2%)
Phenobarbital	31 (21.2%)
Clonazepam	1 (0.7%)
Lamictal	1 (0.7%)
Topiramate	2 (1.4%)

**Table 4:** Curve coordinates.

Cut off Value	Sensitivity	1-Specificity	Specificity
≥-1	1	1	0
≥0.5	1	0.844	0.156
≥1.5	1	0.705	0.295
≥2.5	1	0.566	0.434
≥3.5	1	0.377	0.623
≥4.5	1	0.287	0.713
≥5.5	1	0.213	0.787
≥6.5	1	0.156	0.844
≥7.5	0.917	0.107	0.893
≥8.5	0.875	0.066	0.934
≥9.5	0.792	0.016	0.984
≥10.5	0.625	0.008	0.992
≥11.5	0.5	0	1
≥12.5	0.417	0	1
≥13.5	0.333	0	1
≥14.5	0.25	0	1
≥16	0.125	0	1
≥18	0.042	0	1
≥20	0	0	1

**Table 5:** Diagnostic accuracy assessment.

GAD	GAD (MINI ICD-10)		
(GAD-7)	Yes	No	Total
Yes (≥7)	24	19	43
No (<7)	0	103	103
Total	24	122	146

Sensitivity = a  : (a+c) = 24  : (24+0) = 24  : 24 = 100%

Specificity = d  : (b+d) = 103  : (19+103) = 103  : 122 = 84,4%

Positive Predictive Value (PPV) = a  : (a+b) = 24  : (24+19) = 24  : 43 = 55,8%

Negative Predictive Value (NPV) = d  : (c+d) = 103  : (0+103) = 103  : 103 = 100%

**Table 6:** Clinical characteristics and their relations to GAD (with GAD-7).

	Presence of GAD				
Variables	Yes	No	*p*	OR	95% confidence interval
	n (%) / Median (min-max)	n (%) / Median (min-max)			Lower-Upper
*Age (years)*	36 (18-67)	36 (18-77)	0.812*∗*		
*Age of Onset (years)*	18 (1-62)	16 (1-74)	0.558*∗*		
*Epilepsy Duration (years)*	11 (0.5-44)	11 (0.5-55)	0.811*∗*		
*Seizure Frequency in the last 3 months*	1 (0-46)	1 (0-52)	0.331*∗*		
*Gender*					
(i) Female	20 (29.9)	47 (70.1)	0.922*∗∗*	1.036	0.507 – 2.115
(ii) Male	23 (29.1)	56 (70.9)			
*Education*					
(i) High School Graduate	32 (29.4)	77 (70.6)	0.966*∗∗*	0.982	0.434 – 2.223
(ii) Not High School Graduate	11 (29.7)	26 (70.3)			
*Employment*					
(i) Employed	15 (24.2)	47 (75.8)	**0.231** **∗** **∗**	0.638	0.305 – 1.334
(ii) Unemployed	28 (33.3)	56 (66.7)			
*Marital Status*					
(i) Married	21 (30.0)	49 (70.0)	0.889*∗∗*	1.052	0.516 – 2.144
(ii) Unmarried	22 (28.9)	54 (71.1)			
*Seizure Type*					
(i) Focal Onset	41 (28.7)	102 (71.3)	**0.207*ǂ***	4.976	0.439 – 56.385
(ii) Generalized Onset	2 (66.7)	1 (33.3)			
*Seizure Focus*					
(i) Right	17 (27.9)	44 (72.1)	0.551*∗∗∗*	1.221	0.586 – 2.545
(ii) Left	18 (30.0)	42 (70.0)		1.018	0.490 – 2.116
(iii) Focal Bilateral	5 (33.3)	10 (66.7)		0.851	0.272 – 2.661
(iv) General	2 (66.7)	1 (33.3)		0.208	0.018 – 2.363
(v) No Data	1 (14.3)	6 (85.7)			
*Epilepsy Syndrome*					
(i) Temporal	26 (27.1)	70 (72.9)	0.262*∗∗∗*	1.595	0.743 – 3.428
(ii) Extratemporal	14 (35.0)	26 (65.0)		0.732	0.335 – 1.603
(iii) General	2 (66.7)	1 (33.3)		0.208	0.0.18 – 2.363
(iv) No Data	1 (14.3)	6 (85.7)			
*Epilepsy etiology*					
(i) Genetic	2 (66.7)	1 (33.3)	0.272*∗∗∗*	0.201	0.018 – 2.278
(ii) Unknown Etiology	16 (25.4)	47 (74.6)		1.416	0.683 – 2.939
(iii) Structural	25 (31.3)	55 (68.7)		0.825	0.402 – 1.693
Hippocampal sclerosis/atrophy	10 (27.0)	27 (73.0)	0.450*∗∗*	1.446	0.554 – 3.774
Other structural abnormalities	15 (34.9)	28 (65.1)			
*Number of AED*					
(i) Polytherapy	15 (29.4)	36 (70.6)	0.994*∗∗*	0.997	0.473 – 2.103
(ii) Monotherapy	28 (29.5)	67 (70.5)			
*Type of AED*					
(i) Valproate	15 (35.7)	27(64.3)	0.291*∗∗*	1.435	0.669-3.077
(ii) Carbamazepine	10 (21.7)	36 (78.3)	**0.166** *∗∗*	0.564	0.250 – 1.274
(iii) Levetiracetam	2 (20.0)	8 (80.0)	0.724*ǂ*	0.579	0.118 – 2.847
(iv) Phenytoin	15 (34.9)	28 (65.1)	0.352*∗∗*	1.435	0.669 – 3.077
(v) Clobazam	7 (22.6)	24 (77.4)	0.344*∗∗*	0.64	0.253 – 1.622
(vi) Phenobarbital	11 (35.5)	20 (64.5)	0.406*∗∗*	1.427	0.615 – 3.308
(vii) Clonazepam	0 (0)	1 (100)	1*ǂ*	0.703	0.633 – 0.782
(viii) Lamotrigine	0 (0)	1 (100)	1*ǂ*	0.703	0.633 – 0.782
(ix) Topiramate	1 (50)	1 (50)	0.504*ǂ*	2.429	0.148 – 39.738

*∗*Mann-Whitney *ǂ*Fisher's exact test

*∗∗*Pearson Chi-square

*∗∗∗*Kruskal-Wallis

## Data Availability

The data used to support the findings of this study are available from the corresponding author upon request.
